# 1306. Early Transition to Oral Antibiotics, Including Fluoroquinolone Therapy, for *Streptococcus milleri* Empyema Following Video-Assisted Thoracoscopic Surgery

**DOI:** 10.1093/ofid/ofab466.1498

**Published:** 2021-12-04

**Authors:** Anais Ovalle, Ahmad Alsalman, Timothy Millington, Richard A Zuckerman

**Affiliations:** 1 Dartmouth-Hitchcock Medical Center, Lebanon, New Hampshire; 2 darmouth hitchcock, lebanon, New Hampshire

## Abstract

**Background:**

Pleural empyema from *Streptococcus milleri *(*SM*) is often complex and requires a combination of surgery and intravenous (IV) antibiotics. There is a paucity of data on the efficacy of oral (PO) treatment due to concerns about the development of resistance, particularly to fluoroquinolones (FQ). We report outcomes of postoperative antibiotic treatment for *SM *empyema over 3 years, including PO therapy.

**Methods:**

A single-center retrospective chart review was performed of 20 patients treated with video-assisted thoracoscopic surgery (VATS) from October 2015 to March 2018 and *SM *diagnosed by thoracentesis or operative culture. We reviewed clinical factors, route and duration of antibiotics, complications (empyema recurrence, repeat surgery, 30-day readmission due to empyema), and mortality (30-day and 1-year)

**Results:**

Of the 20 patients, 12 (60%) received all IV and 8 (40%) transitioned to PO therapy (Table 1). Median age was 60 and 58 in the IV and PO group, respectively. IV treated patients had more comorbidities. Cultures were primarily monomicrobial. Isolates tested were susceptible (S) to penicillin (Table 1), Of 10 tested specimen, all had moxifloxacin MIC < 0.19 μg/mL

and 8/8 specimens tested were S to levofloxacin. The average duration of antibiotic therapy in the IV group was 34 days and 32 days in the PO group. There were no complications in the IV group: however, there were 2 deaths (1 patient died from comorbid complications and 1 patient was readmitted and died due to *MSSA* endocarditis). There were no complications or deaths in patients treated PO.

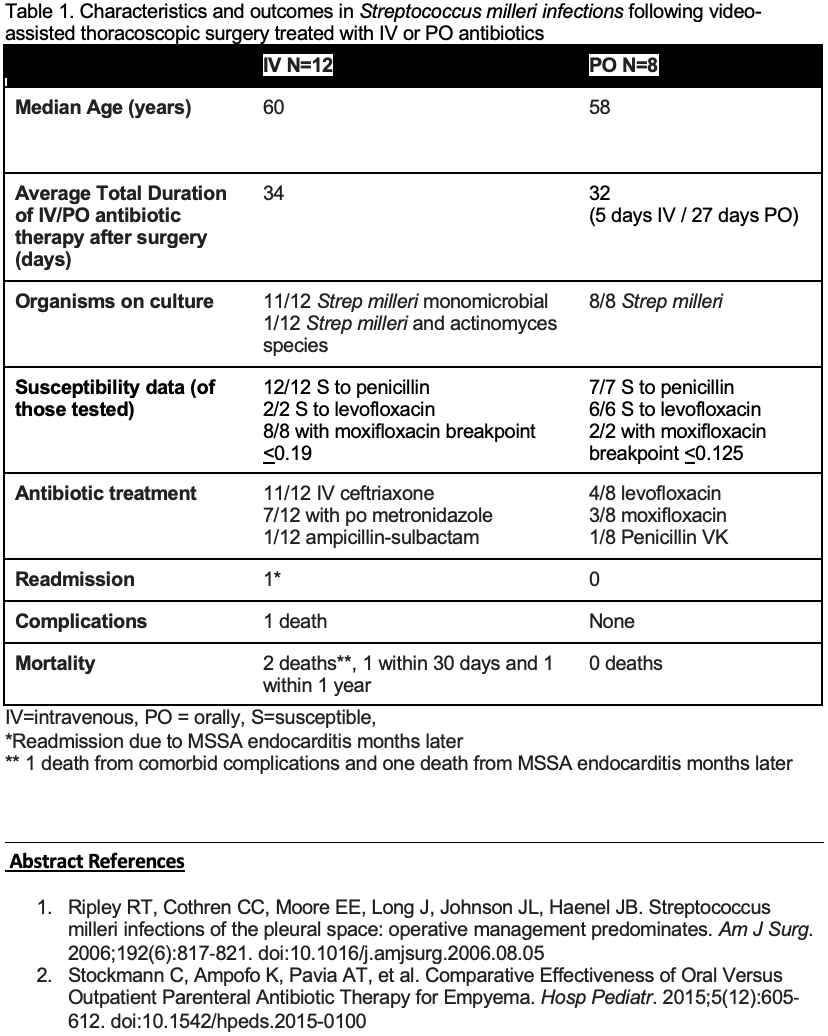

**Conclusion:**

Our review suggests that early transition to PO antibiotics may be a viable option for operatively managed empyema caused by *SM* in certain patients. FQs have been generally avoided due to concerns about the rapid development of resistance that has been shown *in-vitro*; however, no *in-vivo* data have been reported regarding this concern. We show excellent outcomes with the use of PO therapy in susceptible isolates, particularly FQs, with no failure or reported resistance in patients with *SM* empyema treated with VATS. Further study is needed to validate these findings and determine optimal patient characteristics for transition to PO therapy.

**Disclosures:**

**All Authors**: No reported disclosures

